# Bibliographical Notices

**Published:** 1853-01

**Authors:** 


### BIBLIOGRAPHICAL NOTICES.

*A System of Operative Surgery; based upon the Practice of Surgeons in the United States, and comprising a Bibliographical Index and Historical Record of many of their Operations for a period of two hundred years.* By Henry H. Smith, M. D., &c. &c. *Illustrated by numerous steel plates— Parts 1st, 2d, 3d, 4th and 5th* (complete in one vol. 8vo., pp. 698, including index, and 80 plates.) Philadelphia, 1852: Lippincot, Grambo & Co.

In the publication of parts 4th and 5th of Dr. H. H. Smith's Operative Surgery, our readers will be glad to find the completion of his work. We congratulate the author upon the termination of his arduous task. He has good reason to rejoice with all concerned at the very handsome appearance of the volume of colored plates, in which the result of his labors has at last been presented to the public. We cannot discover that there has been any falling off in the preparation of the concluding portions, which it is our business now to notice. On the contrary, we are inclined to think that some improvement has been gained, at least in the coloring of the plates, if not in their general execution.

Part IV. is devoted to Operations on the Genito-Urinary Organs and Rectum. It is divided into five chapters, containing in all about one hundred and four pages, and illustrated by sixteen plates. The first of these chapters is occupied with operations on the male genito-urinary organs, and treats in different sections successively of the surgical anatomy of the parts concerned, of operations on the penis, on the urethra, on the spermatic cord, and on the testicle. Chapter 2d has for its subject, stone in the bladder; and in this connection it presents us with the surgical anatomy of the male perineum, and with the different operations of lithotomy. Chapter 3d discusses the operation of lithotripsy; and in chapters 4th, 5th, 6th and 7th, we find, in regular order of succession, descriptions and representations of operations on the female genito-urinary organs, operations for vaginal fistula, operations practised on the deep seated reproductive organs of the female, and lastly, of operations practised on the rectum.

Part V. concludes the work with an account of operations on the extremities, in eight chapters and eighty-six pages, these latter being accompanied with sixteen beautifully engraved and colored plates. Chapter 1st of this part gives us, under the caption of general operations on the extremities, some observations on the operations for inverted toe-nail, for the cure of paronychia, for the cure of enlarged bursæ, for the relief of certain conditions of the nerves, onthe management of varicose veins, and on the operations of tenotomy. Chapters 2d and 3d describe the ligature of the arteries of the upper and lower extremities; chapters 4th and 5th treat of operations on the bones of the two extremities. Chapter 6th is occupied with general remarks on amputation; and chapters 7th and 8th are devoted to the particular amputations.

Of the thirty-two plates belonging to the concluding chapters just enumerated, the first four are occupied with the instruments employed in operations on the urethra, and with operations practised on the penis, urethra, and the testicle and cord. Then we have five plates devoted to the instruments and operative procedures required for the removal of urinary calculi, including, of course, the usual modes of operating by lithotomy and lithotripsy. Next in order comes a plate which represents a great variety of instruments employed in operations upon the vagina and the rectum. This is followed by four plates, intended to represent the different operations upon the female genito-urinary organs; by two plates exhibiting the operations practised on the rectum; by four plates, in which are shown the deligation of the arteries of the two extremities; by two plates displaying the different resections of the bones of the extremities; and lastly, by one plate presenting the instruments for amputation, and by nine, in which are figured the several amputations of these extremities.

So much for the topics of text and illustration which constitute the material of Dr. Smith's concluding parts. The very brief summary which alone we have been able to offer in this place, will serve, we trust, to show that they are fully equal, in the variety and interest of their contents, to their predecessors already presented to our readers. A careful scanning of their pages, although, as a matter of course, bringing to our notice much that must meet with general approval, and more or less about which there might be a difference of opinion, has nevertheless suggested very little that was possessed of novelty or unusual interest enough to authorize any special comment. It may suffice to note in passing, that of the many well known operations with which the names of American surgeons are connected, he presents us in this portion of his work, among others, with figures and accounts of those for lacerated female perineum, introduced and successfully performed by Mettauer and Horner, for vesico-vaginal fistula by Hayward, Mettauer and Marion Sims, for recto-vaginal fistula by Rhea Barton, for ovariotomy by McDowell and W. L. Atlee, for Cæsarian section by Gibson, for extirpation of the uterus by Esselman and P. F. Eve, for encysted rectum by Physick, for excision of hemorrhoids by Horner, for fistula in ano by Gibson, for anchylosis by Rhea Barton and Gurdon Buck, Jr., and for the introduction of the seton in false joint or ununited fracture by Physick.

Under the head of perineal fistula we are glad to observe a description of the mode of operating which Dr. W. E. Horner has for a long time employed and recommended, but we cannot help regretting that the whole subject of stricture and perineal disease and injury is so hastily dispatched. The management of the prostatic and other portions of the male urethra involved in these perineal dyscrasies is so vitally important and has been of late so frequently discussed, that a few meagre paragraphs and a single operative procedure would hardly seem a fair proportion of attention to it in a systematic work which, notwithstanding the disclaimers of its preface, is manifestly offered as the first attempt at an embodiment of American Operative Surgery, and in which far less interesting questions are allowed to occupy a greater space. The same objection might be made, without injustice, and in some instances with much stronger reason, to other portions of our author's pages, in the earlier as well as later parts. Some of these short-comings are doubtless the result of haste in preparing for the press; or, as intimated in the preface, they could not easily be avoided in a book of plates without an inconvenient development of bulk; some of them arise, perhaps, from mere inadvertence of revision

—to which, by the by, must be attributed the lack of grammatical elegance and perspicuity of language observable in many places. But admitting, with a judgment of charity, that such accidents are excusable, and may not materially impair the standing of an authoritative work, we are still forced to conclude that some of his omissions and superficial modes of treating certain subjects are intentional, and the deliberate result of his particular views. What, for example, are we to suppose of the state of American aural operative surgery if we are to judge from the following cavalier disposal of it in the remarks with which the section on operations on the ear concludes?

"The almost universal necessity that exists in the United States for every surgeon to practise several distinct portions of his profession, as well as the absence of definite instruction in these complaints, usually noticed in the ordinary courses of education of our medical schools, has, for many years, induced the majority of the profession to shun the treatment of aural complaints, and forced patients into the hands of empirics. All the operations upon the ear are, however, so easily practised, and the character of the complaints requiring them so very limited, that this condition of things may be remedied by any surgeon. [!]

"In order to prove this, an effort has now been made to describe, as fully as is necessary, all the ordinary operations required for the relief of deafness, and if the reader will follow the description, in connection with the plates, he will, it is hoped, find them quite full enough. Pages have been written on most of these operations, but with the tendency to confuse and embarrass rather than encourage the reader. Washing out the external and internal auditory tubes, with perforation of the membrana tympani, or perhaps the mastoid cells, really constitutes the entire portion of aural operative surgery, and are certainly easily executed. [!!] The prognosis of the complaints requiring these operations is, it is true, often doubtful, or decidedly unfavorable; yet, it should be remembered that, even when unable to cure, a surgeon may effect much good by assuring the patient of the impossibility of his being relieved, and every operator should, therefore, gain such an amount of practical skill as will enable him to give an opinion. By washing out the meatus externus, and examining the condition of the membrane of the tympanum; by catheterizing the Eustachian tube, or by perforating the membranum tympani, and testing the permeability of the passage to the throat, as above described, much advantage will often be gained by the patient, whilst many persons will be saved from the hands of unprincipled men, who, in the majority of cases, only do them harm." [!!!] (pp. 219, 220.)

We do not believe in the necessity for exclusive devotion to the study of this or of any other department of surgical practice; and we would be sorry to admit the existence of such a lamentable state of ignorance in "the majority of the profession," whether among specialists or not, in regard to diseases of the ear, as the foregoing extract would lead us to imagine.

Again, under the head of means of radically curing reducible hernia, we are treated to the following bright light on an old question.

"After the reduction of a hernia and the application of a truss, the patient is secure for the time from the danger of strangulation, and though it has been asserted that radical cures have been effected by the constant use of the instrument, inducing such adhesions and induration of tissue as plugged up the ring, my opportunities (and they have not been slight) have never enabled me to see one well grown adult who had obtained this result from the use of an instrument. In children and young persons, such a condition has been created as prevented the reproduction of the complaint for years; yet, even in these patients, the success has been far from constant.

"The most, therefore, that can be asserted of any truss is, that after its application, the patient is not liable to a descent of the hernia, provided it fits well, and is constantly worn. The manufacture and application of these instruments having, in many sections of the country, passed into the hands of ignorant men, professional evidence of the advantages resulting from the use of any particular kind of truss is rare. Some, I think, do more harm than good, and the surgeon should, therefore, make it his duty to examine the mode in which the truss is worn, as the instrument is often so badly adapted to the part as to increase the complaint.

"It being generally admitted that little or no reliance can be placed upon a truss for the accomplishment of the radical cure of hernia, several surgeons have endeavored to find some other means of effecting this important object. Most of these, though differing in the details, have had one grand object in view, and that is, the creation of such a condition of the parts as would effectually and permanently close the opening." (p. 413.)

This free and easy way of solving our doubts as to the radical cure of hernia by trusses, does not happen to agree with very respectable evidence against his confident assertion in our own country, and certainly does not accord with the very positive experience of European surgeons, as may be easily ascertained by reference to the works of authors of repute on both sides of the channel.

Once more; in the concluding paragraphs of his remarks on the lateral operation he thus disposes of the Gorget question.

"There are, however, many surgeons in the United States who do not use the gorget, preferring a beaked knife, of various shapes, most of which are apparently favorites, from having been designated by the name of the inventor. In many instances, such knives are only poor modifications of a gorget; they act in the same manner, but do not make so accurate an incision, and are liable to create an opening in the pelvic fascia by leading the operator to incise the prostate to too great an extent laterally. In a deep perineum, it is always difficult to judge of the position of the point of a knife, even when apparently directed by the left fore-finger; but with a staff held in the median line of the body, with its curve close under the pubis, and with the beak of a gorget well placed in it, it is impossible to extend an incision beyond the limits of the width of the blade. This subject is, however, one which has engaged powerful advocates on both sides, and I shall, therefore, dismiss it with the simple statement of individual preference for the gorget of Physick, though, at the same time, I should not hesitate to cut for stone with a staff and pocket bistoury, if nothing else could be obtained, nor doubt the possibility of a surgeon operating neatly and properly with any instrument when a correct anatomical knowledge of the structure concerned was made to direct it." (pp. 522, 523.)

Fortunately for the guidance as well as reputation of American surgeons, this same question, so far as substitutes for the gorget are concerned, is more fully, as well as modestly, discussed by the only other recent American writer—one who certainly enjoys the most extended reputation, at home and abroad, to say nothing of his numerous operations, and his long course of practice and teaching as a surgeon of the highest standing. Yet, although an opponent of the gorget, Dr. Gross, to whom we allude, does not allow us to suppose that even with his authority he would thus contemptuously treat the instrument which, old-fashioned and almost disused as it is, Dr. Smith chooses to style the type of all—the one *par excellence*—of which the different forms of knife constantly preferred by the first surgeons in the world are only so many poor modifications.

These quotations have been taken as we happened to be struck with them in looking through the book. We might add many others equally characteristic; but there is enough already to acquit us of unfairness in expressing the opinion which, as conscienscious reviewers, we are forbidden to withhold, that our author does not afford such a "comprehensive view of the opinions, operative methods and instruments of those of his countrymen who have given to American surgery a character of its own," as the language of its title and preface would imply to be the grand object of his work.

We have not the least desire to quarrel with his individual opinions in regard to the theory and practice of his art, so long as they are allowed to rest solely upon his personal responsibility, and to go for what they may be individually worth. But we cannot aid in presenting them to the professsion under the guise of a national authority, or even the prestige of a strictly native origin. Let the "system" to which they are attached be avowedly established on the "basis" of their author's individual opinions and experience, and not upon that of the "views of his own countrymen," or of the practice of American surgeons in general, and we will bow to his decisions with all the deference due to the superior advantages and opportunities which his seventeen years' devotion to his favorite branch has given to him. We are aware of the tendency of the "sovereigns" of our democracy to imagine their doings and sayings to be American, merely because they happen themselves, as individuals, to belong to the American nation. We know that we are all too apt, in our republican simplicity, to mean "I, the people," when we say "we, the people"; and our only hope in connection with this new competitor for national distinction is, that strangers as well as natives may not fall into the common error. Our friend's book, however creditable to himself and advantageous to us all, cannot afford us an adequate idea of the actual state of surgery on this side of the Atlantic. This could not be expected in its limited amount of space for letter press, and with its independent doctrines in regard to different subjects; while the very large preponderance of European illustrations and the number of operative precepts—nearly all the amputations for example—taken directly from the French, (see p. 665,) give it rather the aspect in some parts of a Parisian compilation. The materials for a genuine cis atlantic work may yet be had abundantly within the reach of those amongst us,—and there should be many such—who are competent and not afraid to use them. The task, however discouraging it may now appear, must sooner or later be fulfilled, without this resort to the enervating draughts from foreign sources, which of late years have been the curse of so many of our publications. No man need go to Paris or London now to learn how to amputate a limb or extirpate a tumor, resect a bone, extract a calculus—in short, to effect the greatest triumph which surgical art and science, under Providence, have ever yet achieved. Why, then, this never-ending slavery to the parasitic feebleness which will scarcely allow us, without resorting to an unknown tongue, to show our pupils how to perform the simplest operation?

Let us not be understood in these remarks to be harboring any prejudice against the work before us, because it happens, from the predilections of its author and his peculiar opportunities, to savor more of European continental surgery than we are inclined to regard as needful in the United States. We have no intention to criticise his taste or judgment as singular in this particular connection, for we admit that he has only followed a prevailing fashion; and we can, and most cheerfully do, recommend his work, whether original and national or not, as the handsomest and most convenient atlas and illustrated outline of operative surgery that has yet appeared in this country, and therefore, one that must prove in a high degree valuable to students, teachers and practitioners. We can recommend it, too, as desirable and important in its influence on account of its many detailed reports of operations by American Surgeons, and of its bibliography; although this latter might have been made a little more complete in several places, without injury to the rest. But we do not venture to endorse it as an epitome or mirror, to use a popular term, of the operative surgery of our own land. Nor, do we mean to say that it is offered, or will be anywhere received as such; and we only have suggested the idea at the risk of making what may seem to be an invidious and gratuitous distinction, in order to clear ourselves, if possible, from the imputation which our notice of the first and second parts has placed us under of favoring such a view. We extended a cordial welcome, in spite of some misgivings at the outset, to an undertaking which bid fair to prove one of the most extensive that had ever been ventured on by an American publisher; and our regard and respect for the enterprise and energy of all concerned led us then, as they do now, to wish them the most flattering success. But we cannot leave them without expressing the disappointment which we sincerely feel, not only at the want of truly native hue in the compilations of the text, but at the almost exclusive preference manifested, in letter press as well as illustrations, wherever foreign aid is resorted to, for French works (and especially the superficial manuals,) to the more practical and substantial productions of the British press. The book, as a specimen of printing and engraving, is most attractive in its getting-up, however; and, although it does not accomplish all that we would like to see effected by such a publication, it still goes far beyond anything we have yet known in its class, and gives us much more, perhaps, than, according to ordinary calculation, we had a right to expect.

---

*A Practical Treatise on Diseases of the Skin.* By J. MOORE NELIGAN, M. D., M. R. I. A., Lecturer on the Practice of Medicine in the Dublin School of Medicine, &c. &c. Philadelphia: Blanchard & Lea, 1852. 12mo. pp. 333.

The Diseases of the Skin constitute an exceedingly important department of nosology, of which, with comparatively few exceptions, practitioners of medicine are lamentably ignorant. This want of knowledge does not depend upon any deficiency of publications upon the affections in question, for such are abundant in the English, French, and German languages, and many of them are accompanied with more or less faithful pictorial representations of the various eruptions described. The difficulty is ascribable partly to peculiarities connected with the diseases themselves, and partly to confusion in the modes of their classification and description. To most physicians they certainly present no special attractions; but, on the contrary, much that is repulsive, in their frequently unsightly appearance, and the not uncommonly disagreeable odor which they occasion. Moreover, they are usually the concomitants, and indeed frequently the result of poverty and filth, and consequently can contribute less to the social position or to the emoluments of the physician than most other complaints which are more equally visited upon all classes of mankind. Again, most of them do not so much interfere with the ordinary pursuits of their victims, as to induce them to resort early to means of cure. And in this country, particularly, there being no public institution for the especial treatment of skin diseases, clinical instruction upon them has never been attempted in any regular and methodical manner; hence young men enter upon the active practice of their profession with the most imperfect notions concerning the diagnosis, and with very empirical views about the treatment of them.

The learner might commence the study of these affections with more hope and zeal, did he see a greater degree of harmony prevailing among dermatologists with reference to the important point of diagnosis. But, inasmuch as the very same disease wears at different stages an entirely different aspect, it has received one appellation from this writer, and another from that; so that at the very threshold of his inquiries the student is perplexed and disheartened.

The two different bases for the classification of cutaneous diseases which are now most prominently presented, are the *artificial* and the *natural*, as they are termed. The first depends upon the specific form and appearances which the eruptions exhibit; the other upon the anatomical seat of the eruption, or, upon the immediate pathological lesion or modification of structure which it indicates. The first was proposed, as Dr. Neligan informs us, by Riolanus, revived by Plenck, and received currency and character from Willan, whose classification, in its essential features, is to this day regarded as the best adapted to facilitate the recognition of the diseases of the skin. The natural systems of classification are chiefly those of Wilson and Cazenave. Mr. Wilson adopts as the basis of his arrangement the anatomy and physiology of the skin, and makes four primary groups of the eruptions which appear upon the latter, viz:

1. Diseases of the Derma.

2. " " Sudoriparous glands.

3. " " Sebiparous glands.

4. " " Hair and hair follicles.

Each has several sub-divisions. M. Cazenave includes all cutaneous affections under eight classes, each of which is founded upon some pathological modification of the structure or properties of the skin, viz:—1, Inflammation; 2, Lesions of Secretion; 3, Hypertrophies; 4, Deteriorations; 5, Hæmorrhagiæ; 6, Lesions of Sensibility; 7, Foreign Bodies; 8, Diseases of the Appendages, embracing the hair and the nails.

If scientific accuracy alone were desirable in a nosological arrangement, as in the classifications of Natural History, it must be confessed that the system of Wilson, which starts from known and always verifiable data, would be the best for cutaneous diseases. But in the examination of these, facility of application must be an important consideration in determining what mode of classification to adopt, and in this respect we cannot but think that the method of Willan possesses advantages over any "*natural*" arrangement yet proposed. It was originally open to serious objections, of course; but it has been of late years so much improved as to be not more amenable to criticism than those which have been advanced to supercede it. The chief objection urged against it is that the characteristic form of the eruption is subject to such changes,—the papule becoming vesicular, the vesicle pustular, &c., from accidental circumstances,—that it may be placed in every group but the right one. It rarely happens, however, that a careful examination will fail in detecting its proper and specific form.

The treatise of Dr. Neligan, which we have already cited by its title, advocates the Willanean system, with the modifications and improvements to which we have just alluded. The author says, "In proceeding to describe the classification of diseases of the skin which I intend to adopt, I wish, *in limine*, to disclaim any pretensions to originality. My chief object is to endeavor to simplify a subject which has often not received from the student and practitioner the attention it merits, owing to the difficulties with which complicated arrangements and even changing nomenclatures have invested it. I shall therefore take advantage of the labors of those who have preceded me, and endeavor to reduce the grouping together of cutaneous eruptions to as few sub-divisions as attention to accuracy will admit."

The arrangement which he adopts is exhibited in a tabular form, as follows:

| Order. | | Genera. |
|---|---|---|
| 1. Exanthemata, | comprising | Erythema, Erysipelas, Urticaria, Roseola. |
| 2. Vesiculæ, | " | Eczema, Herpes, Pemphigus, Rupia, Scabies. |
| 3. Pustulæ, | " | Acne, Impetigo, Ecthyma. |
| 4. Papulæ, | " | Lichen, Prurigo. |
| 5. Squamæ, | " | Psoriasis, Pityriasis. |
| 6. Hypertrophiæ, | " | Icthyosis, Molluscum, Stearrhœa, Elephantiasis, Verruca, Clavus, Callositates, Condylomata, Nævus. |
| 7. Hæmorrhagiæ, | " | Purpura. |
| 8. Maculæ, | " | Vitiligo, Ephelis. |
| 9. Canerodes, | " | Lupus, Keloïs. |
| 10. Dermatophytæ, | " | Porrigo, Sycosis. |

*Supplementary Groups.*

Syphilides,
Diseases of the appendages of the Skin.

The simplification is more conspicuous in the diminished numbers of the varieties included in each of the genera.

Thus, Erythema comprehends, in the arrangement of Dr. Neligan, three varieties: E. simplex, E. papulatum, and E. nodosum. Erythema simplex is made to embrace the E. fugax, E. læve, E. intertrigo, E. marginatum, and E. circinnatum of some other writers,—varieties which depend for the shades of differences which they exhibit upon some peculiarity of the skin in individuals affected, or upon the localization; but which do not present features so striking as to demand a specifie designation.

Of *Acne* he makes but two varieties, calling them after Willan, A. simplex and A. rosacea, rejecting as not deserving special designation the other two varieties of the same author, A. punctata and A. indurata.

Again, he indicates but two varieties of Ecthyma, E. acutum and E. chronicum, instead of the more numerous varieties described by Willan and others, and which are separated from insufficient motives, as the age of the patient, the color of the eruption, and the constitutional cachexia.

In like manner, we might pass over all the orders and genera included in the classification of Dr. Neligan, and show that in almost all of them his pruning-knife has been judiciously exercised in lopping off supernumerary branches which can bear no good fruit for the cultivator of this field of pathological inquiry. We think that in general his modifications are improvements.

The descriptions which he gives of the different affections are sufficiently clear and precise, and the rules for diagnosis will materially assist in the determination of the order and genus to which any doubtful eruption should be ascribed.

He combines in his account, so far as can conveniently be done, the advantages of the natural and artificial system of classification, by not only describing the specific form and appearances of each eruption, but by also designating its anatomical seat and its pathological character. The descriptions are short and are in a great measure pathological definitions.

The treatment recommended is evidently the result of the author's own experience, for it is advised decidedly, and very generally accompanied with an assurance of its utility. The treatment of diseases of the skin is fast losing the empirical character which so long tainted it, when the materia medica contained but three or four articles which any one thought of using in the treatment of cutaneous affections,—Sulphur, Tar-ointment, and Arsenic, to which might be added, from the practice of some one more ambitious than his fellows, mucilaginous baths. Were a physician of the old school of practitioners, one who had not kept himself informed of the improvements, introduced into the various departments of medicine, and particularly into the therapeutics of diseases of the skin, to be suddenly shown the long list of medicinal agents now to be chosen from, he would be struck with amazement at the change.

This department of the subject has occupied a large share of Dr. Neligan's attention, and to it he devotes perhaps the most considerable portion of his book. The details of the treatment of each eruption are given in immediate connection therewith; and in addition to this, the concluding chapter of the volume is consecrated to a review of the general and special therapeutics of cutaneous diseases. The reader will here find a great deal of valuable information upon this subject, together with numerous formulæ for lotions, baths, and for internal administration. The author's recommendations are based upon sound pathological and physiological views, and possess the essential requisite of being the results of his own careful observations. They are comprehensive in their bearing; having regard not to the mere name of the disease, but to the state and stage of the eruption, the peculiar departure from the normal condition of the skin at the part affected, and also of internal organs, and the constitutional condition.

To the beginner, and to the physician who wishes a useful and not voluminous manual to assist his examinations in cutaneous affections, we can highly recommend Dr. Neligan's book. Those who wish to study fully this interesting and neglected class of diseases must, of course, possess themselves of more extended treatises.

We regret to have observed, in perusing this volume, that the American reprint bears evidence of having been hastily passed through the press. It contains very many typographical errors, which could have been easily avoided by greater care on the part of the *employés* of the printing office.

---

*A Memoir of the Life and Character of* JAMES B. ROGERS, M. D., Professor of Chemistry in the University of Pennsylvania. By JOSEPH CARSON, M. D., Professor of Materia Medica and Pharmacy in the University of Pennsylvania. Delivered by request of the Faculty, October 11, 1852, and published by the Class.

This elegant Memoir is a tribute in every way worthy of the subject; distinguished alike by the unaffected simplicity and refined earnestness, which were so eminently characteristic of the lamented colleague of the biographer.

There was much in the career of the late Dr. Rogers to elicit sympathy—especially in his long unrewarded struggles and premature demise, almost immediately after he had reached the goal; and Dr. Carson has delineated it with his proverbial good taste, and all the warmth of long attachment and close intimacy.

The life of Dr. Rogers offers points of much interest to the young aspirant for medical honors. He was emphatically a self-made man. And, when he finally attained the honorable and important post, which he filled for so brief a space, his promotion was strikingly the result of merit unaided by influence; and his selection by the Trustees, and approval by the Faculty of the University, was confessedly but the registration of "the earnest wishes in his behalf and the partiality of the profession."

Dr. JAMES B. ROGERS was born in Philadelphia, in 1803. He graduated in Medicine in 1822, at the University of Maryland, and, almost from the commencement of his professional career, devoted himself to the speciality of chemistry. His first appearance as a teacher was in the chair of Chemistry, in the Washington Medical College of Baltimore. From 1835 to 1839, he occupied a similar position in the medical department of the Cincinnati College. Upon the close of that institution, he returned to Philadelphia, and was soon afterwards engaged in the Philadelphia Medical Institute, where he continued for several years to lecture on the same branch. In 1846, he filled the chair of Chemistry in the Franklin Medical College, of this city, and in

1847, upon the resignation of Professor Hare, he was chosen, "after a spirited canvass," with great unanimity to succeed him. He filled this situation for five years with the highest popularity; when, shortly after the close of the last session, he fell a victim to an insidious disease, which, it appears, had been gradually but surely fixing its hold upon his constitution.

The subjoined feeling allusions of Dr. Carson to the moral attributes of his friend and colleague, will, we know, be responded to with singular warmth and unanimity by the profession and community of Philadelphia:

"He was an object of affectionate regard to those who knew his social worth. Disinterested and generous in his relations with the world, mild and conciliating in deportment, open and affable when approached, urbane to every one, his virtues shone conspicuously within the circle of his friende. With his pupils he was sympathizing; he entered cheerfully into their discouragements and difficulties; and those who confided to him, received that encouragement and counsel so grateful to the student's feelings. He was emphatically the student's friend. In his death, a sore bereavement has befallen us; and we may be permitted to give utterance to our sentiments of sorrow. The grave may conceal from view his mortal lineaments, but they are engraven upon our recollection. A portraiture, distinct and vivid, with all the freshness that affection can give to it, will ever be before our mental vision."

